# Erratum to: “Mismanagement” oder “misunderstanding”?

**DOI:** 10.1007/s00399-016-0480-0

**Published:** 2016-12-12

**Authors:** Helmut U. Klein

**Affiliations:** Heart Research Follow-up Program, University of Rochester Medical Center, Box 653, 265 Crittenden Blvd., 14620 Rochester, NY USA

**Erratum to:**

**Herzschr Elektrophys 2016**

**DOI 10.1007/s00399-016-0470-2**

The article “Mismanagement” oder “misunderstanding”?, written by Helmut U. Klein, was originally published electronically on the publisher’s internet portal (currently SpringerLink) on 10 November 2016 without open access.

With the author(s)’ decision to opt for Open Choice the copyright of the article changed on [24. November 2016] to © The Author(s) [2016] and the article is forthwith distributed under the terms of the Creative Commons Attribution.

